# Case Report and Literature Review of Conjunctival Melanoma in the Black Population

**DOI:** 10.1155/crop/5578922

**Published:** 2025-02-14

**Authors:** Oscar Chen, Fae Kayarian, Elaine Kelly, Richard J. Grostern, Anjali Tannan

**Affiliations:** ^1^Department of Ophthalmology, Rush University Medical Center, Chicago, Illinois, USA; ^2^Rush Medical College, Rush University Medical Center, Chicago, Illinois, USA; ^3^Williams Eye Institute, Hammond, Indiana, USA; ^4^Williams Eye Institute, Crown Point, Indiana, USA

**Keywords:** black, conjunctival melanoma, ocular surface tumor

## Abstract

**Purpose:** The purpose of this study is to report a rare case of conjunctival melanoma (CM) in a black male patient and provide a comprehensive literature review of all documented cases of CM in the black population.

**Methods:** A case report highlighting a black patient with newly diagnosed CM was described. A comprehensive literature review was conducted to determine the prevalence of CM in the black population.

**Results:** Extensive CM in situ in a 46-year-old black male patient was treated with excision and cryotherapy. The patient subsequently required repeat cryotherapy and topical Mitomycin C therapy due to a recurrence of pigmentation. A literature review identified 46 cases of CM in the black population.

**Conclusions:** CM has rarely been observed in the black population. With this current case report, there are only 47 black patients found in the literature with this ocular surface tumor. Further detailed documentation on the presentation, location, and outcomes of CM in this population is imperative to better screen and treat this demographic.

## 1. Introduction

Conjunctival melanoma (CM) is a pigmented neoplastic lesion of the ocular surface. CM comprises 2%–5% of all ocular tumors and 5%–7% of ocular melanoma [1]. The mortality rate of CM can be as high as 30% as it is the most malignant of the conjunctival tumors [2]. The incidence of CM is low given its rarity, with one study demonstrating only 1147 cases in the United States over a 20-year period [3]. CM is even more rare among the black population, with only a few cases reported in the literature. We present a case of CM in a black male as well as an updated literature review on the incidence of CM in this population.

## 2. Patient Consent

The study was conducted in accordance with the tenets of the Declaration of Helsinki. Consent was obtained from the patient to publish the details of the case. The Rush University Medical Center Institutional Review Board acknowledged that the current study did not meet the definition of human subject research. Therefore, no submission for an Institutional Review Board application in the Rush Research Portal was required. The current project was “Acknowledged” with no further action needed.

## 3. Case Presentation

**Figure 1:**
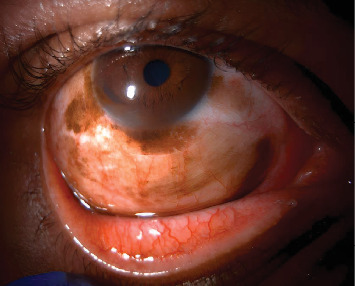
Slit-lamp photography of the right eye demonstrating diffuse pigmentation with involvement of the cornea, bulbar conjunctiva, forniceal conjunctival, and palpebral conjunctiva.

A 46-year-old black male with no significant past medical history presented with pigmentation of the right conjunctiva that had been changing in appearance over the past year. The best corrected visual acuity was 20/20 bilaterally, with normal intraocular pressure and reactive pupils. Extensive heterogeneous pigmentation was noted on the superior bulbar conjunctiva with counterclockwise extension to the nasal conjunctiva (12:00 to 3:00 o'clock). Furthermore, pigmentation on the bulbar conjunctiva extended downward to the inferior fornix and inferior palpebral conjunctiva, with uniform thickness throughout the lesion. Lastly, the pigmentation extended onto the peripheral cornea at 12, 9, and 6 o'clock (Figure 1), with a biopsy excised that measured 4 × 2 cm. The posterior fundus examination was unremarkable, and lymphadenopathy was not appreciated on palpation. Due to the extent and location of the pigmentation, there was a concern for CM.

**Figure 2:**
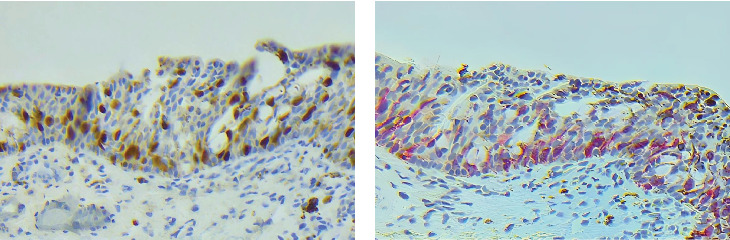
Histology of the resected pigmented conjunctiva. (a) Sox10 and (b) Melan-A stain demonstrating the pagetoid spread of atypical melanocytes throughout the conjunctival epithelium.

Initial evaluation with anterior-segment optical coherence tomography (OCT) did not show invasion of pigmentation into deeper ocular structures. The patient subsequently underwent excision biopsy, double freeze–thaw cryotherapy, superficial keratectomy with absolute alcohol, and amniotic membrane graft placement. The resected margins did not demonstrate any histological findings suggestive of malignancy, indicating the melanoma was excised with clear margins. Histopathological assessment with Melan-A and Sox10 staining demonstrated pagetoid spread of atypical melanocytes within the conjunctival epithelium, confirming the diagnosis of melanoma in situ (Figure 2). The patient was referred to the oncology department for further evaluation. Systemic imaging was unremarkable for metastatic lesions. The patient subsequently underwent a second round of cryotherapy followed by three rounds of topical Mitomycin C (MMC) 0.04% four times daily for a 1-week interval. Residual pigmentation of the palpebral conjunctiva remained, which had a distribution pattern identical to the initial melanoma lesion, but continued to be stable for 1 year following the last dose of the MMC.

## 4. Discussion

CM is exceedingly rare in the black population. Diagnosis of CM can be difficult in this demographic given the prevalence of complexion-associated melanosis, which can be seen in up to 92.5% of patients [4]. The inability to differentiate between racial melanosis and malignant features results in delayed diagnosis along with higher rates of extracutaneous involvement, resulting in an overall worse prognosis for CM in black patients [5].

With this rare ocular surface tumor rarely seen in the black population, a literature review assessing all documented cases of CM in the black population was conducted. A comprehensive literature review was conducted utilizing online databases including PubMed and The National Center for Biotechnology Information to collect English-only literature, using the keywords “conjunctival melanoma,” “black,” and “patient.” To date, only 47 cases of CM in the black population have been documented in the literature (Table 1) [6–8, 11, 13, 14, 16, 18–25]. Given the variability in how cases are described, it is difficult to determine the specific clinical patterns and trends of CM in this demographic. However, most cases present with the recent growth and pigmentation of the conjunctiva along with bulbar conjunctiva involvement. Treatment varied significantly from topical agents such as MMC drops to exenteration of the orbital content.

We present the 47^th^ documented case of CM in the black population. Documented cases of CM in black patients remain uncommon; however, it is likely that many more cases of CM among black patients are missed or misdiagnosed, resulting in worse outcomes and higher mortality rates. Therefore, continuous reporting of CM within this demographic is needed to further understand its initial presentation, symptomatology, and treatments. This will allow for new screening and diagnostic protocols to be developed to better treat black patients with CM.

## Figures and Tables

**Table 1:** Documented cases of conjunctival melanoma in black patients.

**Article**	**Number of cases**
Ash [6]	3
Jay [7]	1
Welsh and Jhatery [8]	1
Kielar [9]	1
Charles et al. [10]	1
Liesegang and Campell [11]	1
Crawford [12]	1
Miller et al. [13]	4
Folberg et al. [14]	7
Schwab and Green [15]	1
Grossniklaus [16]	5
Kalski et al. [17]	1
Singh et al. [4]	2
Shields et al. [18]	1
Yu et al. [19]	5
Colby and Nagal [20]	1
Nunes et al. [21]	1
Medina Mendez and Singh [22]	1
Lemaitre et al. [23]	1
Fasina and Oluwasola [24]	6
Oh and Kanu [25]	1

## Data Availability

The data that support the findings of this study are openly available in all public repositories that issue datasets with DOIs.
